# Effect of thrombus aspiration on microcirculatory resistance and ventricular function in patients with high thrombus burden

**DOI:** 10.1186/s12872-020-01432-1

**Published:** 2020-03-31

**Authors:** Doni Firman, Amir Aziz Alkatiri, Imammurahman Taslim, Surya Buana Wangi, Raymond Pranata

**Affiliations:** 1 Department of Cardiology and Vascular Medicine, Faculty of Medicine, Universitas Indonesia, National Cardiovascular Center Harapan Kita, Jl. Letjen S. Parman Kav 87, Slipi, Jakarta, Barat 11420 Indonesia; 2 Faculty of Medicine, Universitas Indonesia, Jakarta, Indonesia; 3 Faculty of Medicine, Universitas Pelita Harapan, Tangerang, Indonesia

**Keywords:** Thrombus aspiration, Primary percutaneous coronary intervention, Global longitudinal strain, High thrombus burden, index of microcirculatory resistance, Acute coronary syndrome, Thrombectomy

## Abstract

**Background:**

Studies have not demonstrated consistent outcomes following thrombus aspiration in Primary Percutaneous Coronary Intervention (PPCI). We investigated the relationship between thrombus aspiration and microvascular obstruction as measured using Index of Microcirculatory Resistance (IMR) immediately following PPCI and Left Ventricle Function Improvement measured using Global Longitudinal Strain (GLS) six months following PPCI. Our aim is to determine microvascular obstruction and left ventricle function improvement six months following thrombus aspiration during PPCI.

**Methods:**

This was a single-center, observational, prospective non-randomized study involving 45 patients with thrombus score 4–5 (defined as high thrombus burden) and Thrombolysis in Myocardial Infarction (TIMI) flow of 0–2 who subsequently underwent PPCI. Thrombus aspiration was conducted based on physician discretion. The IMR was measured immediately following the procedure. All patients underwent echocardiography to measure GLS at 24 h, 3 months and 6 months following PPCI.

**Results:**

Thirty-three (73%) patients underwent thrombus aspiration during PPCI and twelve (27%) patients underwent the conventional PPCI. No significant difference in IMR was found between the group that underwent thrombus aspiration and the group that underwent conventional PCI (51.9 ± 41.5 vs 47.1 ± 35.6 *p* = 0.723). TIMI flow after PPCI was worse in thrombus aspiration group (OR 5.2 [1.2–23.2], *p* = 0.041). There was no difference in GLS between two groups at 6-month follow-up (− 13.0 ± 3.4 vs − 12.8 ± 4.6, *p* = 0.912).

**Conclusion:**

This study indicates no benefit of thrombus aspiration during PPCI in reducing either microvascular obstruction or left ventricular function at 6-month follow-up for patients with high thrombus burden. Nevertheless, further studies are required before definite conclusions can be made.

## Introduction

Successful remodeling of epicardial coronary arteries’ patency following prolonged occlusion might culminate in microvascular obstruction (MVO). Furthermore, infiltration and activation of neutrophils, platelets, and deposition of fibrins also contribute to reperfusion-induced microvascular damage and obstruction. Additionally, coronary microembolization by atherosclerotic debris after PCI (Percutaneous Coronary Intervention) may be substantially responsible for clinically observed MVO [1]. Adjunctive mechanical devices have been developed to retrieve thrombus from the infarct-related lesion during PCI in patients with ST-Elevation Myocardial Infarction (STEMI). Distal protection devices have been associated with favourable outcome, these devices include distal occlusion devices and distal embolic filters, as well as with anterograde approaches with manual thrombus aspiration catheters or technically more complex mechanical thrombus aspiration catheters [2].

The Thrombus Aspiration during Percutaneous coronary intervention in Acute myocardial infarction Study (TAPAS) trial found that routinely performed thrombus aspiration yielded better myocardial reperfusion and clinical outcomes at one-year follow-up [3]. Since then, thrombus aspiration has been considered as a gold standard procedure during Primary PCI (PPCI), until recently, after the emergence of ThrOmbecTomy with PCI vs PCI Alone in STEMI (TOTAL) and the Thrombus Aspiration in ST-Elevation myocardial infarction (TASTE) trials result [4]. TOTAL and TASTE trials showed the opposite results [2, 5]. The TOTAL trial has shown that routine thrombus aspiration during PCI for STEMI did not improve long-term clinical outcomes and was potentially linked to a higher risk of stroke at one-year follow up [5]. Furthermore, the TASTE trial revealed that routine thrombus aspiration prior to PCI did not reduce 30-day mortality rate compared to PCI alone among patients with STEMI [2].

Cardiac Magnetic Resonance (CMR) is considered as a gold standard to detect MVO [6], however, this procedure is cumbersome. Index of Microcirculatory Resistance (IMR) can measure microvasculature function by using a pressure sensor/thermistor-tipped guide wire. The IMR has a good correlation with CMR in patients undergoing PPCI [7]. The potential advantages of IMR over current methods for evaluating the microcirculation are its relative ease to perform and interpret, its quantitative nature, independence to the epicardial vessel, and reproducibility [8]. The purpose of this study is to evaluate MVO following thrombus aspiration during PPCI through IMR immediately post-PPCI and left ventricular function measured by global longitudinal strain (GLS) six months after PPCI.

## Methods

### Study design and participants

This trial was a prospective, observational, non-randomized study designed to compare the use of routine manual thrombectomy + PCI compared to PCI alone in patients with STEMI with high thrombus burden. The primary outcome of this study was MVO measured by IMR immediately after PPCI. The secondary outcome of this study was left ventricular function (GLS) measured six months after PPCI. This study was conducted in 2014 at National Cardiovascular Center Harapan Kita, Jakarta, Indonesia.

The inclusion criteria were: patients 18 years old or above with the first STEMI episode within 12 h of symptoms onset, Thrombolysis in Myocardial Infarction (TIMI) flow 0–2 and thrombus score 4–5 prior to PPCI. Thrombus score of 4–5 is defined as high thrombus burden. The exclusion criteria were: prior history of PCI, multivessel disease electively planned for revascularization within 6 months following PCI, cardiogenic shock, atrial fibrillation, pacemaker rhythm, bundle branch block, and assisted ventilation.

### Study protocol and procedure

The protocol was approved by the institution ethical board and was performed in accordance with the Declaration of Helsinki. All patients signed written informed consent and pretreated immediately prior revascularization with 300 mg of aspirin, 100 U per kilogram body weight of intravenous heparin, and 300 mg of clopidogrel. Standard primary PCI was performed. Thrombectomy was performed based on operator discretion using Export catheter (Medtronic Inc., Santa Rosa, CA, USA) and Thrombuster catheter (Kaneka Inc., Japan). Aspiration was performed by more than two passages across the lesion. Subsequently, patients received aspirin, clopidogrel (at 12 months), nitrates, beta-blockers, angiotensin-converting enzyme inhibitors, and statins.

### Angiographic analysis

Three experienced observers visually estimated TIMI flow grade and Myocardial Blush Grade (MBG). Thrombus burden at the lesion site was graded from 0 to 5 according to the thrombus score. Inter-observer coefficients of variation assessed in 50 consecutively selected patients for MBG, TIMI, and blush scores were 1%, 9%, and 4%, respectively.

### Physiological measurements

The use of additional pharmacotherapy including glycoprotein IIb/IIIa receptor inhibitors was based on operator’s discretion. Pressure wire (Radi Medical Systems, Uppsala, Sweden) was introduced to the distal 2/3^rd^ of the artery, after calibration following successful culprit lesion stenting. The method for IMR measurement followed previous study by Fearon et al. [9]. Three millilitres of saline was then injected to culprit vessel for three times at rest, after which the resting transit time were averaged. Intravenous adenosine 140 μg/kg/min was administered through central venous catheter to induce maximal hyperemia. Three millilitres of saline were then injected to the culprit vessel followed by the recording of hyperemic transit times. The mean aortic and distal coronary pressures were recorded during peak hyperemia. IMR in this study was defined as distal coronary pressure divided by flow during peak hyperemia.

### Global left ventricle longitudinal strain

Global left ventricle longitudinal strain (GLS) was assessed using the AFI technique. Longitudinal strain (%) is defined as physiological change in length of the region of interest from end-diastole to end-systole. The formula “Longitudinal Strain (in percentage) = [Length (end-systole) – Length (end-diastole)] / Length (end-diastole) x 100%” was used to calculate longitudinal strain [10].

The subjects were examined with Vivid 7 and S6 scanner (Vingmed ultrasound). Standard views were acquired and stored digitally in DICOM format for offline analysis using EchoPAC software. The left ventricle was then divided into 6 segments (inferior, posterior, lateral, septal, anteroseptal and anterior) in which each was divided into 3 different regions (basal, mid and apical). The images for the studies were acquired at frame rate between 60 and 90 frame/second.

All patients underwent echocardiography examination in 24 h, 3 months and 6 months following PPCI. LV improvement was defined as a negative value difference GLS on follow-up minus GLS in 24 h after PPCI.

### Statistical analysis

Continuous data were summarized as median, mean ± standard deviation (SD), mean/median minimum-maximum; discrete data were presented as a percentage. Continuous variables were then analyzed parametrically using Student’s t-test or non-parametrically using Mann-Whitney U test, based on the normality of data. Chi-square or Fisher’s exact test were used to compare dichotomous data. All statistical tests were two-tailed and a *p*-value < 0.05 was considered significant. Patients who did not complete follow-up were excluded from the final analysis. Statistical analyses were performed with SPSS for Windows v.24 (SPSS, Chicago, Illinois, USA).

## Results

**Fig. 1:**
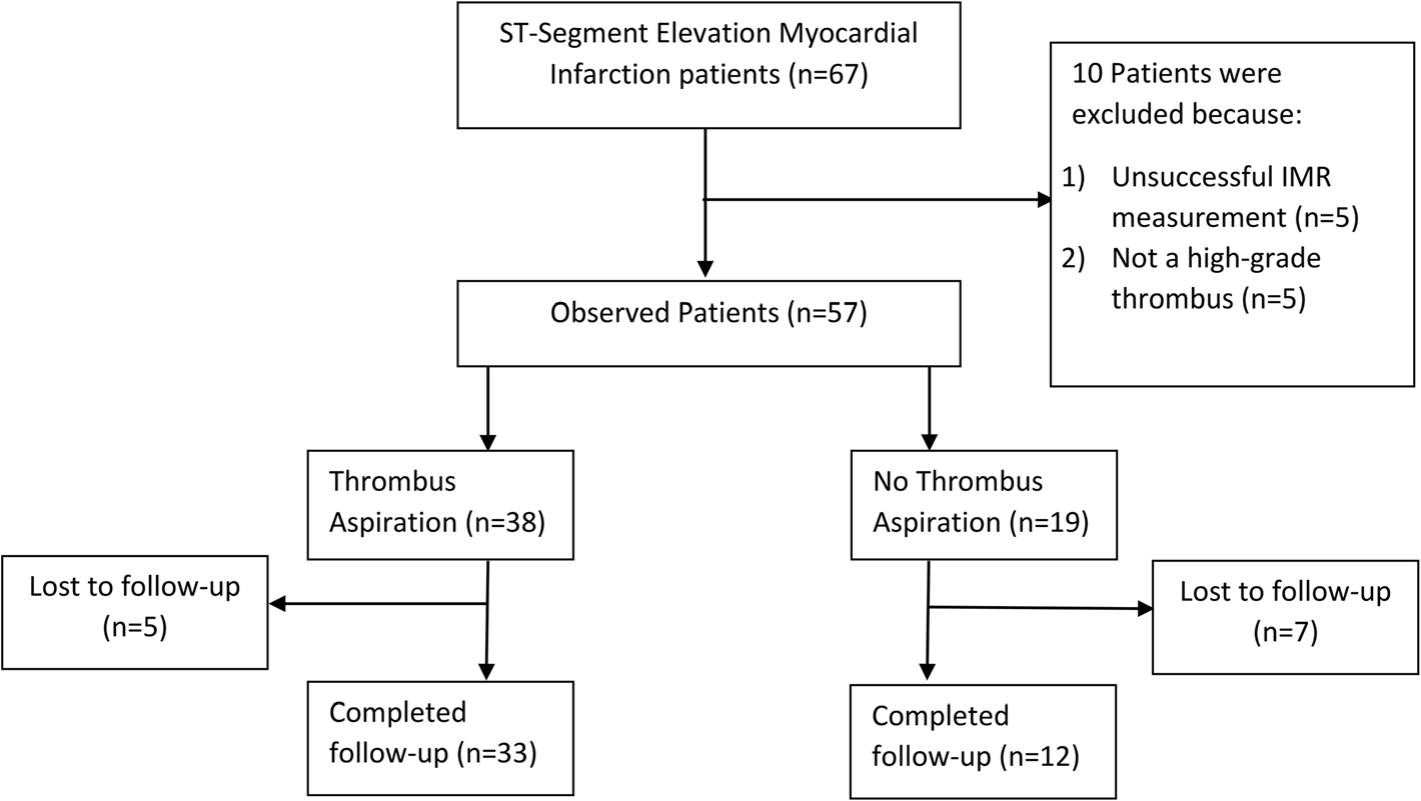
STROBE Flow Chart. IMR: Index of Microcirculatory Resistance

There were 67 consecutive STEMI patients that underwent primary PCI, 10 patients were excluded because of 1) unsuccessful IMR measurement (*n* = 5) and 2) not a high-grade thrombus (n = 5). There were 57 observed patients that fulfilled inclusion and exclusion criteria. 12 patients were lost to follow-up because they did not appear for echocardiography. There were a total of 45 patients that completed follow-up (Fig. 1). The baseline characteristics of the study population are summarized in Table 1. There were no missing data in the 45 patients included in the final analysis. The mean age of the subject was 53.18 years and most patients (91.1%) were men. Thirty patients (66.7%) came with anterior infarction. The mean ischemic time was 423 (343.25–498.8) minutes. The age (*p* = 0.463), ischemic time (*p* = 0.838), door to balloon time (*p* = 0.309), and GPIIb/IIIa inhibitor administration (*p* = 0.246) were similar in both thrombus aspiration and control group (Table 2). TIMI flow 0–2 post PCI was higher in the thrombus aspiration group (OR 5.2 (1.2–23.2), *p* = 0.041). The rate of distal emboli (*p* = 1) and myocardial blush score 0–2 (*p* = 0.491) were similar in both groups. There was no significant difference of IMR between the group that underwent thrombus aspiration and the group that underwent conventional PCI (51.9 ± 41.5 vs 47.1 ± 35.6, *p* = 0.723). There is no significant difference in terms of GLS improvement in thrombus aspiration group compared to control group (p = 1). There is no significant difference in terms of GLS at 6 months follow up using echocardiography (− 13.0 ± 3.4 vs − 12.8 ± 4.6, *p* = 0.912) between the two groups as shown in Table 3. GPIIb/IIIa inhibitor administration was not associated with change in IMR (*p* = 0.847) or GLS at 6 months follow up (*p* = 0.538).

**Table 1:** Baseline Characteristics (*n* = 45)

Characteristics	Value (%) Mean (95% CI)
Male [n (%)]	41 (91.1)
Age (yr.)	53.18 (50.4–56.0)
BMI (kg/m2)	25.26 (24.2–26.3)
Hypertension [n (%)]	16 (35.6)
Dyslipidemia [n (%)]	14 (31.1)
Diabetes mellitus [n (%)]	12 (26.7)
Smoker [n (%)]	36 (80)
Anterior [n (%)]	30 (66.7)
*Door to Balloon Time* (min)	80.08 (67.8–92.4)
Onset of chest pain (hrs.)	6.43 (5.6–7.3)
Ischemic time (min)	423.0 (343.25–498.8)
Stent	1 (0.91–1.1)

Continuous variables are shown in Mean/Median and Range

*BMI* Body Mass Index

**Table 2:** Baseline, Clinical and Procedural Characteristics

Variables	Thrombus aspiration		P
	Yes (***n*** = 33)	No (***n*** = 12)	
Age	54.6 ± 9.7	49.4 ± 7.8	0.463
Male (%)	29 (88)	12 (100)	0.561
Ischemic time (min)	429.8 ± 278.4	404.3 ± 169.2	0.838
Door to Balloon (min)	86.5 ± 34.8	62.3 ± 51.9	0.309
Coronary Flow Reserve	1.24 ± 0.59	1.08 ± 0.39	0.580
Thrombus Burden = 5	29 (93.5)	12 (100%)	1.000
Diabetes Mellitus (%)	9 (27)	3 (25.0)	1.000
Hypertension (%)	12 (36)	4 (25.0)	1.000
Smoker (%)	24 (66.7)	12 (100)	0.086
GPIIB/IIIa inhibitor (%)	9 (27)	1 (8)	0.246
LAD lesion (%)	21 (64)	8 (67)	1.000
Stent	0.97 ± 0.30	1.08 ± 0.28	0.149

Continuous variables are shown in Mean ± Standard Deviation

*GPIIB/IIIa* Glycoprotein IIB/IIIa, *LAD* Left Anterior Descending

**Table 3:** Angiographic, IMR and Echocardiography Results

Variable	Thrombus Aspiration		P	OR (95%CI)
	Yes (***n*** = 33)	No (***n*** = 12)		
TIMI post PPCI				
TIMI 0–2	21 (63.6)	3 (25)	0.041	5.2 (1.2–23.2)
TIMI 3	12 (36.4)	9 (75)		
Distal emboli	2 (6)	0 (0)	1.000	1.4 (1.1–1.7)
Blush score				
(0–2)	13 (39.3)	3 (25)	0.491	1.9 (0.4–8.6)
(3)	20 (60.7)	9 (75)		
IMR (U)	51.9 ± 41.5	47.1 ± 35.6	0.723	
GLS improvement	26 (78.8)	9 (75)	1.000	1.2 (0.3–5.8)
GLS follow up				
GLS 0 months	−11.5 ± 4.8	−12.5 ± 3.3	0.493	
GLS 3 months	−13.6 ± 3.9	−12.9 ± 4.2	0.625	
GLS 6 months	−13.0 ± 3.4	−12.8 ± 4.6	0.912	

Continuous variables are shown in Mean ± Standard Deviation

*GLS* Global Longitudinal Strain, *IMR* Index of Microcirculatory Resistance, *PPCI* Primary Percutaneous Coronary Intervention, *TIMI* Thrombolysis in Myocardial Infarction

## Discussion

 This prospective, non-randomized study indicates that the addition of manual thrombus aspiration to PPCI in patients with high thrombus burden was not associated with benefit in terms of IMR and LV function at 6-month follow-up.

The baseline data showed that most of the patients came with extensive ischemic time (423 min), similar compared to the other developing countries [11, 12]. Lack of awareness of cardiac emergencies among the general public, delayed ambulance services, and difficulties dealing with insurance/financial issues may have contributed to late arrival to the cardiovascular center. Ischemic time in this study was much longer compared to that of TAPAS trial (185–190 min) or TOTAL trial (173–181 min) [4, 5]. This prolonged ischemic time may contribute to the formation of firmer thrombi. Histopathological analysis of aspirated thrombotic content from patients with early ischemic time (less than 12 h) showed erythrocyte-rich (red) thrombi in one-third of patients, predominantly in those presenting with low TIMI flow. A platelet-rich thrombus was identified in the rest of the cases. Analysis of electron microscopic images of thrombi obtained from thrombus aspiration procedures showed that formation of the thrombus was a dynamic process and the composition of the thrombus varied with the ischemic time. Fresh thrombi have the highest proportion of platelets, whereas the proportion of fibrin fibers increased over time leading to older more fibrin rich thrombi [13]. In patients with longer ischemic time (≥12 and ≤ 48 h), the use of thrombus aspiration was not beneficial based on the markers of reperfusion assessed by CMR as compared to conventional PCI [14].

The TAPAS trial showed that the group receiving thrombus aspiration has a better blush scores following PPCI compared to the conventional-PCI only group [4]. Thrombus aspiration prior to stenting resulted in an improved myocardial reperfusion [4]. Myocardial reperfusion was defined as clear improvements in myocardial blush grade and ST-segment elevation resolution, as well as reduction in residual ST-segment deviation [4]. A study conducted by Carlo et al. indicated that thrombectomy (including thrombus aspiration) resulted in better post-procedural ST-segment elevation resolution and reduce in MVO at 3 months [15]. The EXPIRA study also showed benefit of using thrombus aspiration in group with thrombus score ≥ 3 and TIMI flow grade ≤ 1 as represented by MBG after PPCI [16]. The difference between Expira and this study is that we used IMR to determine MVO.

However, the TASTE trial, which compared randomized thrombus aspiration followed by PCI to PCI involving 7244 patients, failed to show any benefit in all mortality causes or any other clinical end-point [2]. The median onset-to-door time in the TASTE trial was 3 h [5, 17], less than half of the time documented in this study. Furthermore, the 3-year cohort study conducted by Jones et al. found no association between thrombus aspiration and the patient’s mortality rate [18]. Besides failing to show any benefit on mortality, the TOTAL trial found that thrombus aspiration increased the incidence of stroke following PPCI [5].

Aligned with the aforementioned established studies, this study also found no significant difference in terms of left ventricular improvement between the two groups. This is comprehensible since the IMR in both groups were high (median 51.9 U for the thrombus aspiration group and 47 U for no thrombus aspiration group)[4]. McGeogh found higher IMR (median [IQR]) among patients with MVO (38 [29 to 55] U) than in patients without MVO (27 [18 to 36] U) [7]. IMR was a negative multivariable predictor of LV ejection fraction and infarct volume on the Contrast enhanced Cardiac Magnetic Resonance (ceCMR) scan two days following MI, and IMR was a multivariable predictor of LV ejection fraction and infarct volume at 3 months [7].

This study discovered that more participants from the thrombus aspiration group has TIMI flow of 0–2 post PPCI compared to those without thrombus aspiration. This finding is unexpected, but presumably due to more abundant distal embolization caused by thrombus aspiration which aggravate the MVO. Thrombus aspiration technique and handling among the operators might also contributed to this finding.

The result of this study also supported the statement of 2015 ACC/AHA/SCAI Focused Update on Primary Percutaneous Coronary Intervention for Patients With ST-Elevation Myocardial Infarction: An Update of the 2011 ACCF/AHA/SCAI Guideline for Percutaneous Coronary Intervention and the 2013 ACCF/AHA Guideline for the Management of ST-Elevation Myocardial Infarction that routine aspiration thrombectomy before primary PCI is not recommended (Class III: No Benefit, LOE A) [19]. There were insufficient data to assess the potential benefit of a strategy of selective or bailout aspiration thrombectomy (Class IIb, LOE C-LD), defined as unplanned thrombectomy performed due to unsatisfactory initial result or procedural complication, analogous to the definition of “bailout” glycoprotein IIb/IIIa use [19]. Recent studies have demonstrated the benefit of complete revascularization [20, 21], nevertheless, at the time this study was conducted, we only performed culprit-only PCI. Hence, this issue was not expected to greatly influence the analysis and its result.

### Clinical implication

This study revealed no benefit of thrombus aspiration during PPCI in reducing either microvascular obstruction or left ventricular function at 6-month follow-up for patients with high thrombus burden. It may be concluded that thrombus aspiration during PPCI might no longer be indicated. Nevertheless, further studies are required before definite conclusions can be made.

### Study limitation

This study was a single-center, observational, non-randomized study with limited number of patients. This especially applies to the non-thrombectomy group. Moreover, cardiovascular core lab was not available. Additionally, thrombectomy is performed at the operator’s discretion may contribute to bias, however, the thrombus burden in the two groups were similar. The follow up time period was only up to 6 months, a longer follow up might be needed. Factors such as myocardial edema may affect the microvasculature and is a potential confounder. Further study involving more subjects in randomized setting is expectedly needed.

## Conclusion

In summary, this study showed that manual thrombus aspiration was not beneficial in reducing IMR and improving LV function at 6-month follow-up compared to PCI without thrombus aspiration among patients with high thrombus burden. Nevertheless, further studies are required before definite conclusions can be made.

## Data Availability

All data generated or analysed during this study are included in this published article. Corresponding author (D.F) can be contacted for more information.

## Abbreviations

AFI: Automatic function imaging
C-LD: C-limited data
CMR: Cardiac magnetic resonance
ceCMR: Contrast enhanced cardiac magnetic resonance
GLS: Global longitudinal strain
IMR: Index of microcirculatory resistance
LV: Left ventricle
LOE: Level of evidence
MBG: Myocardial blush grade
MVO: Microvascular obstruction
PPCI: Primary percutaneous coronary intervention
STEMI: ST-elevation myocardial infarct
TAPAS: Thrombus aspiration during percutaneous coronary intervention in acute myocardial infarction study
TASTE: Thrombus aspiration in ST-elevation myocardial infarction
TIMI: Thrombolysis in myocardial infarction
TOTAL: ThrOmbecTomy with PCI vs PCI alone in STEMI
